# The Impact of Current and Thermal Loads on the Properties of Copper Contact Wires Used in Railway Transport Systems

**DOI:** 10.3390/ma19091854

**Published:** 2026-04-30

**Authors:** Adam Mańka, Krzysztof Aniołek, Andrzej Hełka, Jan Rak

**Affiliations:** 1 Department of Rail Transport, Faculty of Transport and Aviation Engineering, Silesian University of Technology, Krasińskiego 8, 40-019 Katowice, Poland; adam.manka@polsl.pl (A.M.); andrzej.helka@polsl.pl (A.H.); 2 Institute of Materials Engineering, Faculty of Science and Technology, University of Silesia, 75 Pułku Piechoty 1A, 41-500 Chorzów, Poland; jan.rak@us.edu.pl

**Keywords:** copper, contact wire, current-induced heating, microstructure, micromechanical properties, material degradation

## Abstract

The paper presents the results of investigations of the effect of current heating on the microstructure and properties of copper profile contact wires used in railway traction networks. The aim of the study was to determine the effect of the contact wire temperature rising as a result of current heating on the occurrence of permanent changes in material characteristics. The tests were conducted in laboratory conditions through controlled heating of samples with a current of approx. 1500–1600 A, reaching temperatures of up to approx. 280 °C. It was found that in the temperature range of 120–200 °C, changes in the mechanical properties of the material were insignificant, while above 200 °C, a marked decrease in microhardness and a reduction in the indentation modulus were observed. The results obtained indicate that long-term current load leading to an increase in the temperature of the contact wire may cause deterioration of the mechanical properties of the material, its increased susceptibility to permanent deformation and an increased risk of damage to the traction network, which has a direct impact on the durability of contact wires and the maintenance costs of the rail transport infrastructure.

## 1. Introduction

Copper is a material with very favourable properties. It stands out from other metals primarily due to its high electrical conductivity, second only to silver, which is why it is widely used in the electrical engineering and electronics, construction, transport and chemical industries [1,2,3,4]. Furthermore, it is a soft metal with low durability and very good plastic properties. It also has high thermal conductivity. In contrast to the wide range of possibilities offered by copper alloys, the possibilities for shaping the structure and properties of pure copper are much more limited. Strengthening of copper is mainly achieved through cold working processes. However, exceeding the recrystallisation temperature during operation may lead to the loss of work hardening due to the formation of new, undeformed grains. This process may result in a reduction in the hardness and strength properties of the material and an increase in its plasticity. The recrystallisation temperature of copper depends on a number of factors, such as the degree of its chemical purity, the content of impurities, the amount of cold deformation, the degree of processing and the grain size before cold deformation, etc. [5,6,7].

Copper and its alloys are widely used in rail transport, both for the construction of traction network components and for the distribution of electricity in power substations, power distribution panels, transformers and other electrical components [8,9,10]. During operation in a railway environment, these components are exposed to variable temperatures resulting from climatic fluctuations, electric current flow and heating due to friction. These factors can significantly affect the performance and durability of copper components, including contact wires used in rail transport [11].

The railways of the European Union member countries operate electric traction vehicles equipped with pantographs whose heads are fitted with contact strips made of composite (carbon) material. This solution is required by the TSI regulations (Technical Specifications for Interoperability) [12]. The implementation of these requirements necessitated certification testing of materials offered by various manufacturers, carried out by specialised testing units. The results obtained formed the basis for approving the materials for use in contact strips for current collectors. One of the important evaluation criteria was the temperature criterion. During the tests, it was found that in the area where the composite strip met the contact wire, the temperature rose to high values, often approaching the maximum permitted by regulations. This phenomenon was intensified in the case of contact strips co-working with a contact wire with a significant degree of wear, which is of significant operational importance, as such wires are found on many sections of the traction network in use. Numerous publications devoted to the problem of heating at the contact area between the strip and the copper contact wire can be found in the literature [13,14,15,16,17]. The authors of this paper have also conducted numerous studies confirming the problem of high temperatures generated in the area where the composite strip comes into contact with the contact wire [18,19].

Temperature is one of the most important factors affecting the structural stability and performance properties of copper. Elevated temperatures can lead to microstructural changes, such as grain growth or recrystallisation, which in turn causes a deterioration in mechanical properties [20,21]. Repeated thermal cycles, typical for operating conditions in rail transport, may additionally promote oxidation, surface degradation and a decrease in material hardness, which reduces electrical conductivity and increases wear in the contact area [22,23,24]. Analysis of the literature and operational experience have shown that comprehensive knowledge of the effect of temperature changes on the mechanical properties of copper components in real railway conditions is limited. Understanding these correlations is key to ensuring the reliability and safety of electrical and mechanical systems operating in demanding operating environments.

This study determined the impact of excessive thermal load on the microstructure and micromechanical properties of electrolytic copper used in the manufacture of profile contact wires for railway transport systems. The thermal load of the tested copper samples was achieved by passing a current of approx. 1500–1600 A through them. The results obtained may contribute to the development of operational strategies related to the maintenance of traction networks and electric traction vehicles in modern railway transport systems.

## 2. Materials and Methods

**Figure 1:**
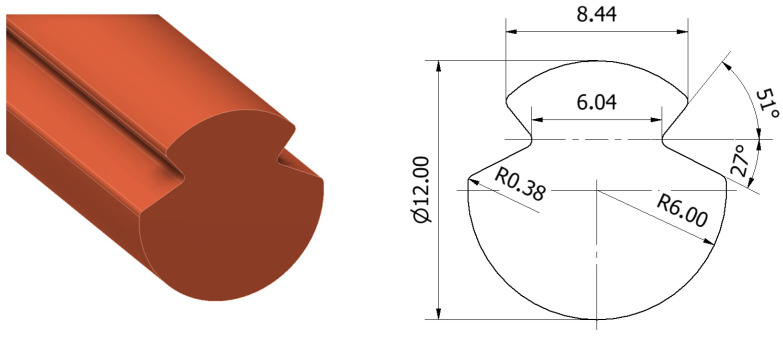
Spatial model and geometric dimensions of the Djp100 profile contact wire.

As part of this study, thermal tests were conducted on electrolytic copper of the Cu-ETP grade (Cu 99.9%), compliant with the EN 1977:2000 standard and the guidelines contained in EN 50149:2012 and PN-E-90090:1996 [25,26], used for the production of profile contact wires in rail transport. The tests utilised Djp100 contact wires manufactured by Power Cable Company in Będzin (Poland). The basic geometric, material and electrical characteristics of the tested contact wires are presented in Figure 1 and Table 1.

**Figure 2:**
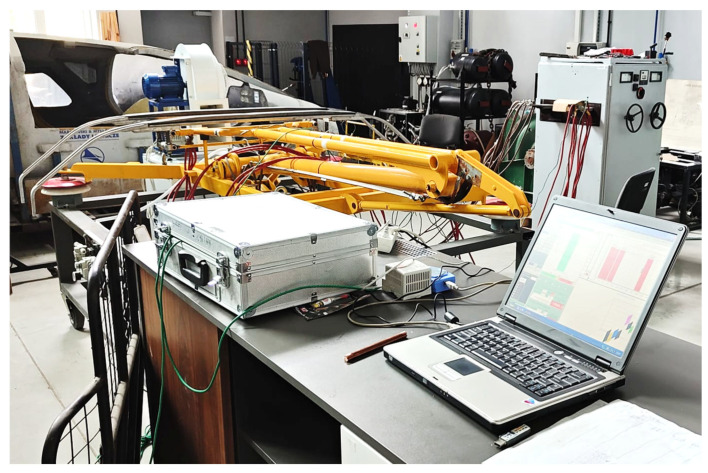
Test stand with a power supply system and 160EC current collector.

The tests simulated the current conditions occurring in real railway operating conditions. The tests were carried out in a laboratory equipped with a power source ranging from 300 A to 2400 A and a voltage of up to 10 V. The experimental station, together with the power supply system and current collector, is shown in Figure 2.

**Figure 3:**
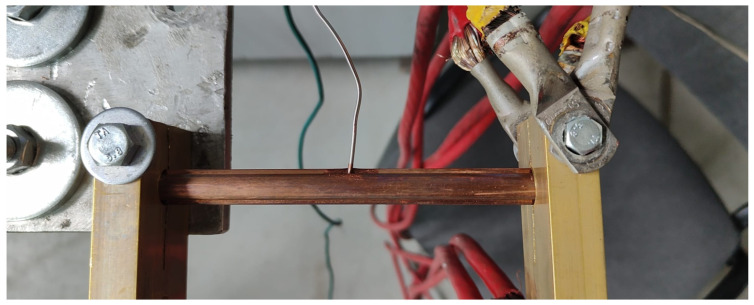
Sample in the current stand holder.

A railway pantograph was connected to the circuit in which the sample was placed (Figure 2). The pantograph acted as an additional resistor in the circuit and was intensively cooled by air. The current flow through the sample was achieved by fixing it in brass holders, to which 16 power supply cables were connected (8 on each side) (Figure 3). Regardless of the ammeter readings on the power supply unit, the current strength was also measured using a Lutron CM-9930 clamp meter (Lutron, Taipei, Taiwan, China) with serial number 04011 and a measurement accuracy of ± 2% A. During the tests, current source setting No. 2/5 was used, which corresponded to a current intensity in the range from 1509 A to 1638 A, depending on the connected receiver. Changes in current intensity resulting from circuit resistance influenced the rate of temperature rise in the system. Slight deviations in current values observed during measurements, caused by intense heating of system components, were compensated for on an ongoing basis by adjusting the power supply source parameters accordingly.

The temperature of the samples was measured using an ICP-CON series I7018 system with type K thermocouples (ICPDAS-Europe, Reutlingen, Germany). The temperature measurements were recorded at a frequency of 1 Hz. Due to the possibility of overheating system components, the temperature of the pantograph and supply cables was also monitored and recorded. The IT-BAS type thermocouples have a measuring range up to 1400 °C and are electrically insulated. The temperature measurement system featured cold junction compensation (separate measurement of thermocouple junction temperatures). A SATIR thermal imaging camera, model SAT-S180, serial number 180010127 (SATIR, Drogheda Co., Louth, Ireland), was used to verify the temperature distribution throughout the sample.

**Figure 4:**
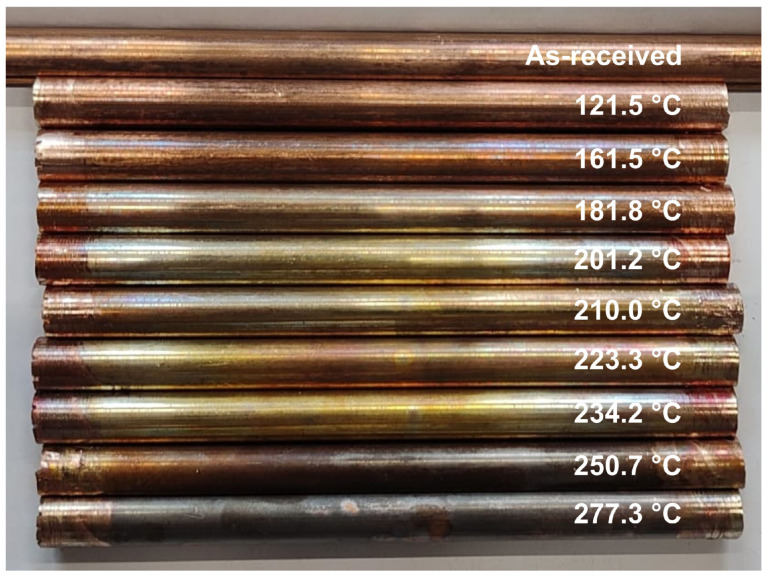
Copper samples after thermal loading with high-intensity current (maximum temperature).

The installation of subsequent samples was preceded by thorough cleaning of the areas to which the jaw holders were connected (Figure 3). In each test, measurements were started at a sample and power supply temperature close to the ambient temperature. Due to the relatively low supply voltage (up to 10 V), even slight changes in contact resistance caused changes in current intensity. During the tests, it was necessary to provide a controlled, gradual increase in the temperature of the sample and immediate shutdown of the power supply system once the temperature specified in the experiment plan was reached. A photograph of the samples after electrical and thermal stress is shown in Figure 4.

In the next stage, samples were prepared for microscopic and micromechanical testing by hot mounting in phenolic resin with carbon filler (PolyFast resin from Struers, Ballerup, Denmark) in a fast-mounting press. Afterwards, grinding was performed using wet abrasive paper with the following grit sizes: 220, 360, 600, 800, 1000, 1200 and 1500. In the next stage, the samples were polished with diamond pastes (MetaDi Monocrystalline Diamond Suspension by Buehler, Lake Bluff, IL, USA) with the following grit sizes: 6, 3 and 1 µm. The samples were subjected to chemical etching using the Mi17Cu reagent, intended for copper and its alloys. This process enabled the revelation of grain boundaries in electrolytic copper, allowing for their further analysis using optical microscopy. The etching solution was prepared in the following proportions: 30 mL of hydrochloric acid (HCl), 10 g of ferric chloride (FeCl_3_), and 120 mL of ethanol (C_2_H_5_OH). The applied etching procedure ensured rapid and effective microstructure development of the investigated material, enabling clear identification of grain boundaries.

Observations of the microstructure of electrolytic copper before and after current tests were performed using an Olympus GX-51 light microscope (Olympus, Tokyo, Japan). The bright-field technique was used for observations. Observations were conducted at 500× magnification. Microscopic images were recorded using the Olympus Stream Essentials software (version 1.7).

Instrumental hardness tests of copper samples before and after electrical and thermal load were performed using a Micro Combi Tester, MCT^3^ (Anton Paar, Corcelles-Cormondrèche, Neuchâtel, Switzerland). The Vickers indenter was used in the study. Indentation curves were recorded at a maximum load of F_max_ = 500 mN. The duration of loading and unloading was determined in accordance with the recommendations of ISO 14577 standard [27]. The hold time under maximum load was 10 s. For each specimen, 8 indentations were made. Based on the recorded indentation curves, the following parameters were determined: hardness H_IT_ and indentation modulus E_IT_.

## 3. Results

### 3.1. Examination of Current and Thermal Loads on Profile Contact Wires

**Figure 5:**
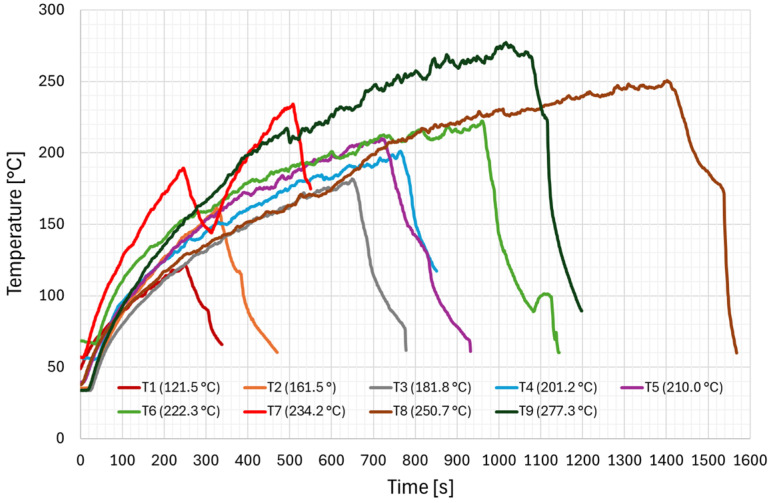
Temperature changes over time for copper samples under current load.

The first stage of the analysis of the test results focused on the temperature curves of the samples depending on the set values of current intensity. Figure 5 shows the temperature changes of copper contact wires during current loading as a function of time, illustrating the process of wire heating and temperature stabilisation during testing. Table 2, in turn, summarises the parameters recorded during the current tests, including the maximum temperatures obtained for individual samples.

Based on the analysis of the results obtained, it was found that the heating speed increased with the increase in current intensity. This phenomenon was observed as a change in the angle of inclination of the curve relative to the time axis (Figure 5). Changes in current intensity were corrected by adjusting the power supply parameters. By doing so, it was possible to ensure a comparable rate of temperature increase during the tests, so that the obtained temperature result was affected as little as possible by the inertia of the measuring system.

The tests showed that the maximum temperatures obtained during the tests ranged from 121.5 °C to 277.3 °C, while the maximum current values ranged from 1487 A to 1638 A. It was found that the temperature of the copper contact wires was mainly influenced by the current flow duration, which ranged from 251 s to 1403 s. As the duration of the tests increased, an increase in the maximum temperature was observed, which is consistent with the resistive heating mechanism, in which the amount of heat generated is proportional to the square of the current intensity, electrical resistance and the duration of its flow. As the heating time increased, thermal energy accumulated in the material, leading to a further rise in temperature. Furthermore, as the temperature rose, the electrical resistance of copper increased, which also led to an acceleration of the heating process, particularly in the final phase of the tests. The test results confirmed that at high current flows (approx. 1.5–1.6 kA), the duration of the load was a key factor in determining the temperature of the contact wire. This means that the temperature of the material was affected not only by the current value, but also by the duration of its flow and the conditions of heat dissipation to the environment.

The conducted tests have shown that when high current flows, the temperature of copper profile contact wires can rise to values exceeding 200 °C, which in traction applications can be considered a potentially dangerous status due to the possibility of deterioration of mechanical properties and deterioration of the conditions of interaction at the pantograph-wire contact area. The research results confirm the need to take into account the effects of heating of contact wires when analysing overload conditions and designing traction power supply systems, especially in situations of increased power consumption or prolonged current load.

**Figure 6:**
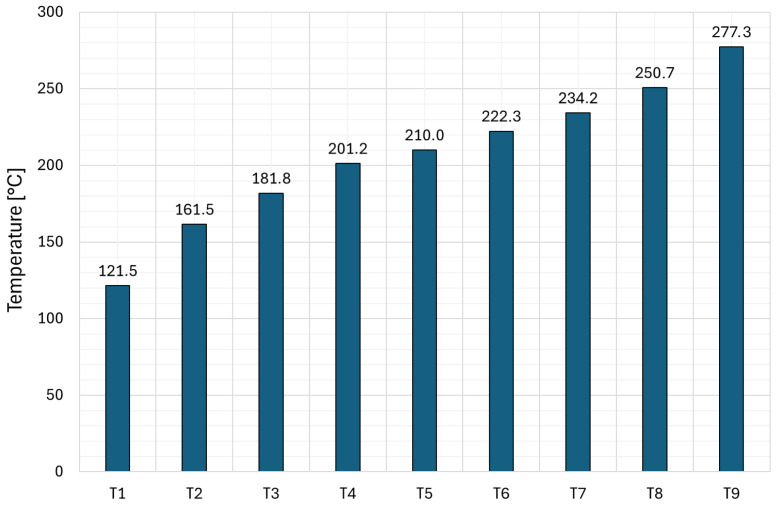
Summary of maximum temperatures obtained during current and thermal testing of copper samples.

Figure 6 shows a summary of the maximum temperatures obtained during current and thermal tests of copper used for profile contact wires. It was found that the average temperature increase between successive samples was 19.5 °C, while the minimum temperature difference was 8.8 °C and the maximum, obtained in the temperature range from 121.5 °C to 161.5 °C, was 40 °C. The highest concentration of measurement points was set in the range of middle temperatures, where material changes were expected to occur.

The analysis of the results obtained allowed for the development of a model of heating of a contact wire at a given load of 1518 A and the determination of the coefficients of the function approximating this process. This dependence can therefore be utilised in the phase of numerical modelling of the contact wire. By approximating the T8 curve (250.7 °C), the following dependence of temperature change was obtained as a function of time:

T(t) = −9.0108·10^−5^·t^2^ + 0.25714297·t + 61.605 (1)

**Figure 7:**
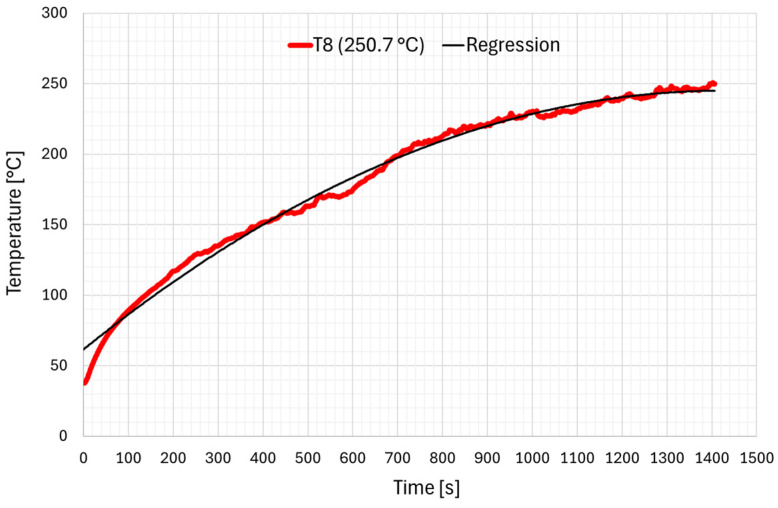
Temperature change over time for sample T8 (250.7 °C) together with the polynomial regression model according to (1).

Figure 7 shows the temperature changes over time for sample T8 (250.7 °C) together with an approximation curve. The regression obtained is characterised by matching with a coefficient of determination R^2^ = 0.9921 for 1403 test seconds. This means that the curve accurately reflects the changes in sample temperature measured during the test.

The developed heating model of the contact wire is based on the measured temperature evolution of the tested sample T8 (Figure 7) as a function of time under a current load of 1518 A. The coefficients of the approximating function were determined using the Ordinary Least Squares (OLS) method, which evaluates the quality of fit between the function and the experimental data. The optimization of the coefficients themselves was performed using the Generalized Reduced Gradient (GRG) method. This is a nonlinear optimization algorithm used to find the minimum of the error function by following its gradient.

It is worth noting that, as expected, the coefficient associated with the t^2^ term is negative, reflecting the increasing influence of convective heat transfer over time. To eliminate the limitation associated with the use of a second-order polynomial, which predicts a decrease in temperature after 1427 s (parabolic behavior), this relationship may instead be described by Equation (2), i.e., the so-called Newton exponential heating curve:

(2) $$T\left(t\right)=T_{P}+∆T\cdot \left(1-e^{-\frac{t}{\tau }}\right)$$

After substituting the constants, the equation takes the following form:

(3) $$T\left(t\right)=40+225\cdot \left(1-e^{-\frac{t}{600}}\right)$$

where:

T_p_—initial temperature [°C];
τ—thermal time constant of the object.

### 3.2. Analysis of the Microstructure of Copper in the As-Received Condition and After Current and Thermal Loading

**Figure 8:**
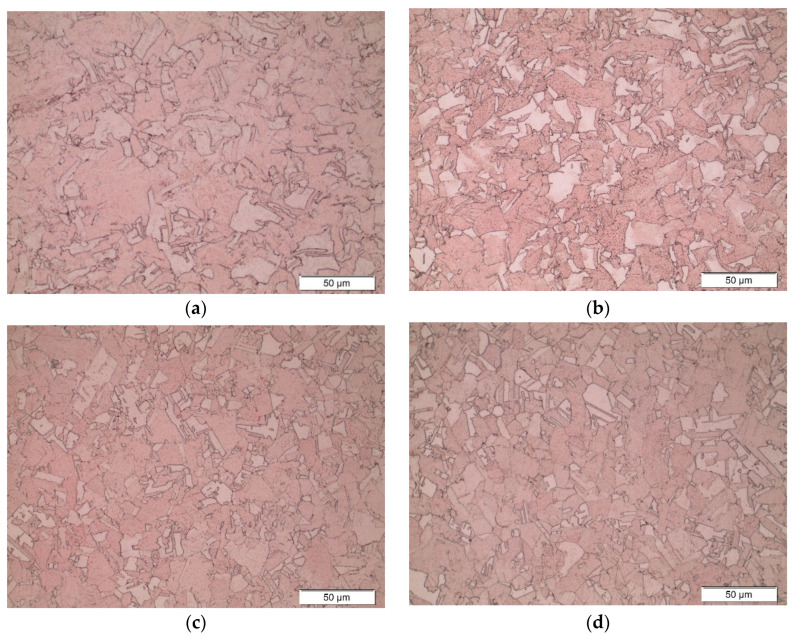
Microstructure of copper in the as-received condition (**a**) and after thermal loading at temperatures of 121.5 °C (**b**), 201.2 °C (**c**) and 277.3 °C (**d**).

Figure 8 shows microscopic images of electrolytic copper Cu-ETP in its as-delivered state and after current and thermal loading, as a result of which the material was heated to temperatures of 121.5 °C, 201.2 °C and 277.3 °C.

The as-received material had a microstructure characteristic of post-annealed copper (Figure 8a). The microscopic image shows grains of varying polygonal shapes with visible grain boundaries. The approximate grain size ranges from approximately 20 to 60 µm. Within the copper grains, the presence of rectilinear twin boundaries can be observed, which is characteristic of materials such as copper. The absence of impurities or pores indicates the high purity of the material.

After current and thermal loading of the contact wire, which led to a temperature increase of up to 121.5 °C, no changes were found in the copper microstructure compared to the as-received material. In this case, polygonal grains with distinct boundaries and a comparable size to those in the initial state (approx. 20–60 µm) were also found. No increase was observed in the grain size, suggesting that the temperature of about 120 °C was too low to initiate microstructural change processes. It was also found that the rectilinear twin boundaries were still clearly visible, indicating that the initial as-annealed structure had been preserved.

Due to thermal loading of the tested material at temperatures up to 201.2 °C and 277.3 °C, the microstructure did not change significantly and remained characteristic of as-annealed copper. No increase in the grain size was observed. Microscopic images show rectilinear twin boundaries within the grains, whose contrast increased and dimensions decreased as the temperature rose. This phenomenon may be related to stress relaxation, local recrystallisation or microstresses caused by heating induced by the flow of electric current [28]. Microscopic observations showed that the microstructure of copper heated to temperatures exceeding 200 °C does not change significantly compared to the as-received material. However, it can be assumed that further increase in the temperature (above 300 °C) will cause more pronounced microstructural changes, in particular an increase in grain size [29,30].

### 3.3. Examination of Micromechanical Properties

**Figure 9:**
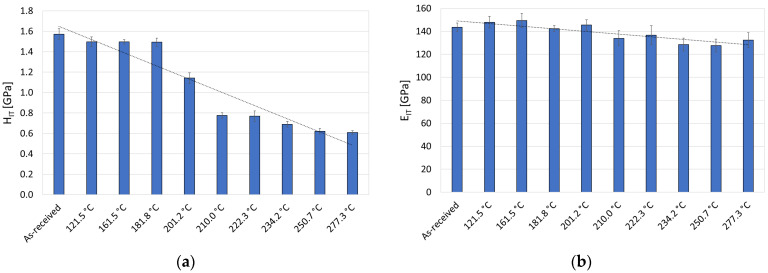
Hardness H_IT_ (**a**) and indentation modulus E_IT_ (**b**) of copper in the as-received condition and after current and thermal loading in the maximum temperature range of 121.5–277.3 °C.

Figure 9 shows the results of micromechanical tests on copper in its as-received condition and after current and thermal loading in the temperature range of 121.5–277.3 °C, obtained at a maximum load of 500 mN and a loading/unloading speed of 16.7 mN/s.

Figure 9a shows the change in the microhardness H_IT_ of copper used for profile contact wires as a function of temperature obtained as a result of currents of approximately 1500–1600 A flowing through it. The hardness of the sample in its as-received condition was approximately 1.55 GPa. After thermal loading in the range of 121.5–181.8 °C, it was found that the changes in hardness were insignificant and comparable to the values obtained for the as-received material. A significant decrease in hardness was observed only after thermal loading of copper at temperatures exceeding 200 °C. It was found that the hardness of H_IT_ decreased with an increase in temperature. For the highest temperature obtained in the tests (277.3 °C), the H_IT_ microhardness was approximately 0.6 GPa, representing a decrease of approximately 61% compared to the as-received material.

Figure 9b shows the change in the indentation modulus E_IT_ of copper used for contact wires as a function of temperature obtained as a result of current and thermal loads. For the material in its as-received condition, the value of the indentation modulus E_IT_ was approximately 145 GPa. Within the temperature range of 160–180 °C, it was found that deviations in the E_IT_ parameter were insignificant (approx. 145–150 GPa). However, above 200 °C, a systematic decrease in the indentation modulus E_IT_ (to approx. 125–130 GPa) was observed. The decreasing tendency of the E_IT_ module is comparable to that observed in the case of hardness, but the changes are less intensive. This means that the processes occurring in the material (stress relaxation, local recrystallisation) also affect its elasticity, but to a lesser extent than its resistance to plastic deformation.

The study demonstrated that the indentation modulus (E_IT_) exhibits significantly lower sensitivity to temperature changes in copper induced by electrical heating compared to indentation hardness (H_IT_), indicating a partial decoupling of the material’s elastic and plastic responses. This phenomenon arises from the different physical mechanisms governing these properties. The elastic modulus is primarily determined by the nature of interatomic interactions, which depend on the type of bonding and the crystal structure of the material. Consequently, the value of the indentation modulus (E_IT_) is relatively insensitive to microstructural changes. In contrast, indentation hardness (H_IT_) is directly related to plastic deformation mechanisms and strongly depends on the microstructure, including dislocation density and grain size (according to the Hall–Petch relationship) [31]. The decrease in copper microhardness (H_IT_) after electrical heating can also be associated with an increased concentration and mobility of crystal lattice defects (vacancies) at elevated temperatures. These defects reduce lattice cohesion and facilitate dislocation motion, leading to a decrease in the material’s resistance to plastic deformation and a deterioration of its mechanical properties [32,33].

### 3.4. Analysis of the Negative Effects of Current and Thermal Loading on Profiled Contact Wires and the Direction of Further Research

The research has shown that the heating of contact wires as a result of traction current flow is significant for their durability and operating reliability. On the basis of an analysis of the experimental test results, it was found that prolonged exposure of contact wires to current of high intensity can lead to self-annealing of the wires as a result of self-heating, which results in a deterioration of, among others, their mechanical characteristics.

Under operating conditions, the heating of copper contact wires can cause many unfavourable effects. As a result of recrystallisation and grain growth, the structure of copper becomes softer and less resistant to abrasion. Furthermore, in paper [11] it was found that the current flowing through the traction wires generates heat, which consequently leads to energy losses and changes in the stresses and bend curves of the suspended contact wires. This also leads to accelerated ageing of the wires, which results in a decrease in the elastic and tensile strength, and consequently reduces the safety margin of the structure. The above factors result in the need for more frequent inspections and replacements of components, and consequently lead to increased costs of maintenance of the traction infrastructure.

In particular, attention should be paid to operating conditions where very high current loads and unfavourable vehicle movement parameters occur simultaneously. In practice, this mainly applies to the moment when a heavy load starts moving from a semaphore, when, in one hand, the currents consumed are reaching very high values, and in the other, the speed of the vehicle is very low, close to 0 km/h. Under such conditions, the contact area between the contact strips of the pantograph and the contact wire heats up significantly. In addition, the situation is worsened by increased use of the contact wire in these critical areas, which contributes to a further increase in temperature. Elevated temperatures cause increased susceptibility of copper to plastic deformation and creep, as well as a decrease in its hardness. As a result, the contact wire becomes more prone to permanent elongation under the influence of tensile loads and its own weight. The recurrence of such unfavourable situations in the same areas, for example in the vicinity of semaphores, may lead to gradual weakening and, in extreme cases, even damage to the traction network.

Further research should focus on determining the impact of load cyclicality and verifying whether frequent current and thermal loads cause further gradual degradation of the material. This issue is particularly significant when a train comes to a stop and then resumes movement from the same place on the track, generating cyclical, locally concentrated loads at the same points or on the same section of the contact wire. Another important issue is determining the temperature distribution in the contact wire in the area of its contact with the contact strip of the pantograph. Obtaining information about the spatial distribution of temperature in a Djp-type contact wire and assessing the effectiveness of heat dissipation mechanisms, such as conduction, convection and radiation is crucial for the design of contact strips, as well as for determining the acceptable current values in the conditions where a train is stationary or in the start-up phase.

## 4. Conclusions

The conducted research has confirmed reports in the referenced literature concerning the gradual degradation of the mechanical properties of technically pure copper used for profile contact wires in the railway traction network during intensive operation. This issue is also indicated in frequent operational reports concerning cases of traction network breakage due to excessive current load, occurring especially in places where heavy freight train units start moving, e.g., before semaphores. This phenomenon is often observed in the case of Djp100 contact wires, which are characterised by a significant degree of wear. It is a serious matter because it concerns many sections within the operating traction network.

The aim of the study was to determine the impact of the contact wire temperature which increases as a result of heating by the flow of current, on the potential for permanent changes in material characteristics, forming the basis for further research into cyclic loads. A model of the heating of the contact wire under a given load has also been developed, which may be used for numerical modelling in the future.

The research has shown that the flow of high-intensity current, which causes heating of copper profile contact wires, leads to significant changes in the mechanical properties of the material depending on the temperature obtained. In the temperature range of 120–200 °C, the observed changes were insignificant, which indicates that the mechanical properties of the material remain relatively stable in this temperature range. Distinct changes in the properties were only observed after exceeding the temperature of circa 200 °C, when a significant decrease in the microhardness of the material was observed. At the highest temperature obtained during testing (approx. 277.3 °C), the hardness value decreased by approximately 61% compared to the as-received material.

The results obtained indicate that prolonged current loads leading to an increase in the contact wire temperature above 200 °C can cause a significant deterioration in the mechanical properties of the material, which under operating conditions may contribute to increased susceptibility of the wire to permanent deformation, increased sag of the contact wire and an increased risk of damage of the traction network. These changes are therefore significant for the rate of contact wire wear during traction network operation and indirectly affect the maintenance costs of the rail transport infrastructure.

## Figures and Tables

**Table 1:** Basic properties of Djp100 profile contact wires.

Nominal Cross-Section of Contact Wire [mm^2^]	Nominal Weight of Contact Wire [kg/km]	Minimum Tensile Strength [MPa]	Minimum Elongation [%]	Maximum Resistance of Contact Wire at 20 °C [Ω/km]
100	890	355	3	0.183

**Table 2:** Summary of sample designations and test parameters.

Series Name	T1	T2	T3	T4	T5	T6	T7	T8	T9
Max. temperature [°C]	121.5	161.5	181.8	201.2	210.0	222.3	234.2	250.7	277.3
Test time [s]	251	326	650	766	720	960	509	1403	1018
Max. current [A]	1545	1573	1528	1607	1490	1627	1638	1518	1487

## Data Availability

The original contributions presented in the study are included in the article. Further inquiries can be directed to the corresponding author.
